# Associations Between Severity of Depression, Lifestyle Patterns, and Personal Factors Related to Health Behavior: Secondary Data Analysis From a Randomized Controlled Trial

**DOI:** 10.3389/fpsyg.2022.856139

**Published:** 2022-03-18

**Authors:** Alejandra Aguilar-Latorre, Maria J. Serrano-Ripoll, Bárbara Oliván-Blázquez, Elena Gervilla, Capilla Navarro

**Affiliations:** ^1^Institute for Health Research Aragón (IIS Aragón), Zaragoza, Spain; ^2^Research Network in Preventive Activities and Health Promotion (RedIAPP), Barcelona, Spain; ^3^Balearic Islands Health Services, Primary Care Research Unit of Mallorca, Palma, Spain; ^4^Research in Preventive Activities and Promotion and in Cancer Illes Balears (GRAPP-CAIB), Health Research Institute of the Balearic Islands (IdISBa), Palma, Spain; ^5^Department of Psychology and Sociology, University of Zaragoza, Zaragoza, Spain; ^6^Department of Psychology, University of the Balearic Islands, Palma, Spain; ^7^Statistic and Psychometric Procedures Implemented in Health Sciences Research Group, Health Research Institute of the Balearic Islands (IdISBa), Palma, Spain

**Keywords:** lifestyle, sleep quality, physical exercise, diet, personal factors, depression

## Abstract

**Background:**

Depression is a prevalent condition that has a significant impact on psychosocial functioning and quality of life. The onset and persistence of depression have been linked to a variety of biological and psychosocial variables. Many of these variables are associated with specific lifestyle characteristics, such as physical activity, diet, and sleep patterns. Some psychosocial determinants have an impact on people’ health-related behavior change. These include personal factors such as sense of coherence, patient activation, health literacy, self-efficacy, and procrastination. This study aims to analyze the association between the severity of depression, lifestyle patterns, and personal factors related to health behavior. It also aims to analyze whether personal factors moderate the relationship between lifestyles and depression.

**Methods:**

This study is a secondary data analysis (SDA) of baseline data collected at the start of a randomized controlled trial (RCT). A sample of 226 patients with subclinical, mild, or moderate depression from primary healthcare centers in two sites in Spain (Zaragoza and Mallorca) was used, and descriptive, bivariate, multivariate, and moderation analyses were performed. Depression was the primary outcome, measured by Beck II Self-Applied Depression Inventory. Lifestyle variables such as physical exercise, adherence to Mediterranean diet and sleep quality, social support, and personal factors such as self-efficacy, patient activation in their own health, sense of coherence, health literacy, and procrastination were considered secondary outcomes.

**Results:**

Low sense of coherence (*β* = −0.172; *p* < 0.001), poor sleep quality (*β* = 0.179; *p* = 0.008), low patient activation (*β* = −0.119; *p* = 0.019), and sedentarism (more minutes seated per day; *β* = 0.003; *p* = 0.025) are predictors of having more depressive symptoms. Moderation analyses were not significant.

**Discussion:**

Lifestyle and personal factors are related to depressive symptomatology. Our findings reveal that sense of coherence, patient’s activation level, sedentarism, and sleep quality are associated with depression. Further research is needed regarding adherence to Mediterranean diet, minutes walking per week and the interrelationship between lifestyles, personal factors, and depression.

## Introduction

Depression affects an estimated 280 million people globally, making it a leading cause of disability and a major contributor to the global burden of disease [World Health Organization (WHO), 2021]. Also, depression has a significant impact on psychosocial functioning and quality of life (Malhi and Mann, 2018). The onset and persistence of depression have been linked to a variety of biological and psychosocial variables, many of which are associated with specific lifestyle characteristics (e.g., poor-quality diet, sleep disturbances, and sedentary lifestyle; Toobert et al., 2007; Hidaka, 2012; Kupfer et al., 2012; Lopresti et al., 2013). Accordingly, some healthy habits (e.g., good dietary, good sleep quality, and adequate physical activity) are linked to reduced levels of depression (Olivan-Blázquez et al., 2018; Ka-Yan Ip et al., 2021; Wong et al., 2021). Specifically, physical exercise interventions as a treatment for depression appear to have a moderate to large effect (Josefsson et al., 2014; Kvam et al., 2016). Additionally, the severity of depression and current depression diagnosis is associated with an unhealthy dietary intake, poorer dietary quality, and a lower Mediterranean diet score (Oliván-Blázquez et al., 2021). Similarly, there are also associations between mental health and having a healthy diet and a good amount of sleep (Hepsomali and Groeger, 2021). Besides that, depression positively predicted poor sleep quality over time (Wakefield et al., 2019), and likewise, sleep disorders increase the risk of suicidal behavior in depressed patients (Wang et al., 2019).

Furthermore, some personal factors are also connected to mental health. Some of these factors are self-efficacy (Sherer et al., 1982), patient activation in their own health (Hibbard et al., 2004), sense of coherence (Antonovsky, 1993), health literacy (Sørensen et al., 2015), and procrastination (Guilera et al., 2018). First, self-efficacy represents a person’s confidence in their ability to self-regulate specific behaviors when confronted with various obstacles/barriers (Bandura, 1977; Sherer et al., 1982). Self-efficacy facilitates the intention to engage in preventive health behavior (Dominick et al., 2013) and is related to depression (Milanovic et al., 2018). Second, activation in their personal health is a factor present in patients with better physical and mental health, who engage in more frequent individual exercise (Hibbard et al., 2004). Patient activation is negatively associated with depression (Magnezi et al., 2014). Third, sense of coherence (SOC) is a factor that determines how well a person manages stress and stays healthy (Antonovsky, 1993), and its relationship with depression is highly reported (Konttinen et al., 2008; Giglio et al., 2015). Fourth, health literacy reflects an individual’s capacity to independently engage in effective health communication and use health-related resources (Nutbeam, 2000). Individuals with adequate health literacy are more likely to participate in preventive behaviors, have more disease-specific knowledge, and have good health management skills (Dominick et al., 2013). Health literacy and depression correlate negatively (Hsu et al., 2020). Fifth, procrastination is the irrational and voluntary delaying of necessary tasks (Guilera et al., 2018) and is associated with perceived stress, depression, anxiety, and fatigue (Beutel et al., 2016). These personal factors are framed around the theory of salutogenesis (Antonovsky, 1996). The salutogenic approach aims to enhance participants’ mental health and wellbeing by increasing their awareness, confidence, and ability to use their personal factors related to health behavior (Langeland and Vinje, 2016).

In light those previous associations between lifestyles, personal factors, and mental health, this study aims to analyze the association between the severity of the depression, some lifestyle patterns (physical exercise, sleep, and diet), and some personal factors related to health behavior (self-efficacy, activation in their own health, sense of coherence, health literacy, and procrastination). It also aims to analyze whether personal factors moderate the relationship between lifestyles and depression.

## Methods and Analysis

### Study Design

This research project is a secondary data analysis (SDA; Wickham, 2019) of baseline data collected at the start of a randomized controlled trial (RCT; Aguilar-Latorre et al., 2020), whose main objective is to evaluate the effectiveness and cost-effectiveness of a lifestyle modification program in the prevention and treatment of subclinical, mild, and moderate depression in primary care settings.

### Sample Size

The sample size was established in the RCT study (Aguilar-Latorre et al., 2020). A total of 226 participants were included in this study.

### Recruitment and Participants

The selection of participants was done using individuals who consulted a general practitioner (GP) from the participating primary healthcare centers (PHCs) for any reason and who met the inclusion criteria from the RCT study. The inclusion criteria were the following: individuals over the age of 18, either male or female, scoring ≥10 and ≤ 30 points on the BDI-II (Beck et al., 1996), who were experiencing depression symptoms for at least 2 months, could understand written and spoken Spanish, and also had provided their written informed consent. The exclusion criteria were the following: individuals suffering from another disease that affects the central nervous system (organic brain pathology or having suffered a traumatic brain injury of any severity, dementia); individuals with another psychiatric diagnosis or psychiatric severe illness (substance dependence or abuse, history of schizophrenia, or other psychotic disorders, eating disorders) with the exception of anxiety pathology or personality disorders [collected through a medical history and from the Mini-International Neuropsychiatric Interview (MINI; Ferrando et al., 2000)]; individuals with a severe or uncontrolled medical, infectious, or degenerative illness that may have interfered with the affective symptoms; individuals experiencing delirium or hallucinations, risk of suicide, pregnancy, or lactation; patients who had participated in another clinical trial over the past 6 months or who were in psychotherapy; individuals who practiced mindfulness, yoga, meditation, or similar practices for the preceding 6 months, and who engaged in formal training at least once a week; and the presence of any medical, psychological, or social problem that could seriously interfere with the patient’s participation in the study. Recruitment started in April 2020 for a period of 7 months. A sample of 226 patients with subclinical, mild, or moderate depression was recruited from PHCs from two sites in Spain (Zaragoza and Mallorca).

Participants’ data were collected in the PHCs through a structured interview carried out by Research Assistants (RA) who had received specific face-to-face training to ensure the standardization of data collection. Some RAs were involved in coding the data while others conducted the outcome assessments and data analysis. All information collected was treated following the provisions of current legislation on personal data protection.

### Study Variables

Sociodemographic data: We collected information on gender (female, male), age, marital status (without a partner: single, separated, divorced, in separation proceedings, widower or widow; and with a partner: married or living with a partner), education (none or primary and secondary or tertiary), occupation (working: active and not working: unemployment, homemaker, unpaid work, student, pensioner, sick leave, temporary job disability, permanent job disability, and other situations), and economic level (<Interprofessional Minimum Wage (IMW) to 2 IMW and > 2 IMW).

Depressive symptomatology was measured using the BDI-II (Beck et al., 1996). It consists of 21 multiple-choice questions, with each response being graded on a scale ranging from 0 to 3. The validated Spanish version has a Cronbach’s alpha value (α) of 0.89 (Sanz et al., 2005). The standardized cut-offs are 0–13: minimal depression; 14–19: mild depression; 20–28: moderate depression; and 29–63: severe depression. The internal consistency of the BDI-II in our sample was acceptable (*α* = 0.72).

Social support was measured using the Medical Outcomes Study Social Support Survey (MOS-SS; Sherbourne and Stewart, 1991). It has 19 items, with a 5-point Likert scale, measuring four subscales (emotional/informational, tangible, affectionate, and positive social interaction) and an overall functional social support index. Higher scores indicate increased support. The validated Spanish version has good reliability (*α* ≥ 0.91) and has been quite stable over time (de la Revilla-Ahumada et al., 2005). The internal consistency of the MOS-SS in our sample was excellent (*α* = 0.95).

Physical activity was measured using the International Physical Activity Questionnaire-Short Form (IPAQ-SF; Kim et al., 2013). It contains seven items and records the activity over the last 7 days, depending on intensity levels: vigorous-intensity activity, moderate-intensity activity, and walking and sitting. We used the validated Spanish version (Roman-Viñas et al., 2010). The IPAQ-SF had good reliability for vigorous physical activity and sitting hours, poor validity for moderate activity, and moderate reliability for walking (Kurtze et al., 2008). In our analysis, we use the minutes walking per week and the minutes seated per day.

Adherence to a Mediterranean diet was assessed using the 14-item Mediterranean Diet Adherence Screener (MEDAS), developed by the PREDIMED study group (Martínez-González et al., 2010). It includes items related to food consumption and consumption habits, such as the preference for white meat over red meat, portions of vegetables, fruit, red meat or sausages, portions of animal fat, sugar-sweetened beverages, red wine, legumes, fish, commercial pastries, and dressings made with traditional tomato sauce, garlic, onion, or leeks. The total score is between 0 and 14, with a higher score indicating better adherence to a Mediterranean diet (Schröder et al., 2011).

Quality of sleep and sleep patterns were measured using the Pittsburgh Sleep Quality Index (PSQI; Buysse et al., 1989). It distinguishes between “poor” and “good” sleep by assessing seven domains: subjective sleep quality, sleep latency, sleep duration, habitual sleep efficiency, sleep disturbances, sleep medication use, and daytime dysfunction over the previous month. It consists of 19 self-administered questions and five questions requesting the assessment of the patient’s partner or housemate (which are not scored). Responses range from 0 (no difficulty) to 3 (extreme difficulty). The total score is between 0 and 21 points. The Spanish translation has an *α* of 0.81, with a sensitivity of 88.63%, and a specificity of 74.99% (Royuela-Rico and Macías-Fernández, 1997). The internal consistency of the PSQI in our sample was acceptable (*α* = 0.75).

We also assessed personal factors related to health behavior: (1) self-efficacy (Sherer et al., 1982); (2) activation (Hibbard et al., 2004); (3) sense of coherence (Antonovsky, 1993); (4) health literacy (Sørensen et al., 2015); and (5) procrastination (Guilera et al., 2018).

Self-efficacy was measured using the Self-Efficacy Scale (SES; Sherer et al., 1982), which is made up of two subscales: the General Self-Efficacy subscale (17 items assessing the individual’s beliefs about their ability to perform well in various situations) and the Social Self-Efficacy subscale (6 items). It consists of 23 items, each rated on a 14-point scale (ranging from strongly agree to strongly disagree). Higher scores indicate higher levels of self-efficacy. It has an *α* value of 0.86 for the General Self-efficacy subscale and 0.71 for the Social Self-efficacy subscale. Godoy translated the unpublished Spanish version in 1990 (Lopez-Torrecillas et al., 2006). The internal consistency of the SES in our sample was good (*α* = 0.87).

Patient activation in their own health was measured using the Patient Activation Questionnaire (PAM) regarding their health management (Hibbard et al., 2004). It assesses the patient’s perceived knowledge, skills, and confidence to engage in self-management activities. It consists of 23 items, each rated on a 4-point scale. Higher scores indicate higher levels of activation (Hibbard et al., 2004). This scale has been validated exclusively for chronic patients in Spanish. It had an item separation index for the parameters of 6.64 and a reliability of 0.98 (Moreno Chico et al., 2018). The internal consistency of the PAM in our sample was good (*α* = 0.86).

Sense of coherence was measured using the Sense of Coherence (SOC-13) questionnaire (Antonovsky, 1993). It assesses an individual’s willingness to assess vital experiences. In addition, it assesses the sense of coherence, comprehensibility, manageability, and meaningfulness. It consists of 13 items, each rated on a 7-point scale. Higher scores (after reversal of the inverted items) indicate a higher sense of coherence. It has a consistency rate of 0.84 to 0.93. We used the validated Spanish version (Moreno et al., 1997). The internal consistency of the SOC-13 in our sample was acceptable (*α* = 0.78).

Health Literacy was measured using the Health Literacy Europe Questionnaire (HLS-EUQ16; Sørensen et al., 2015). Health literacy refers to individual skills used to obtain, process, and understand health information and the competencies necessary to make appropriate health-related decisions. It contains 16 items, each rated on a 4-point scale. Higher scores indicate worst health literacy. Its validated Spanish version has an α value of 0.98 (Nolasco et al., 2018). The internal consistency of the HLS-EUQ16 in our sample was excellent (*α* = 0.91).

Procrastination was measured using the Irrational Procrastination Scale (IPS; Steel, 2010). To measure general procrastination (the dysfunctional action of delaying or postponing something). It contains nine items, rated on a 5-point Likert scale, with higher scores (after reversal of the inverted items) indicating a higher level of procrastination. It has an *α* value of 0.90. We used the validated Spanish version (Guilera et al., 2018). The internal consistency of the IPS in our sample was good (*α* = 0.83).

### Statistical Analysis

Firstly, a descriptive analysis was performed (frequencies for categorical variables; means and standard deviation for continuous variables) to determine the characteristics of the sample. Secondly, to analyze the associations between the BDI-II score and all the variables, correlations were performed using the Pearson correlation coefficient test. Thirdly, a multiple linear regression was performed (Núñez et al., 2011), using a stepwise method to obtain a better fitting result upon statistical analysis. This stepwise regression simply repeats multiple regression, deleting the least correlated variable each time (Hamilton and James, 1994). Only the significant variables obtained in the bivariate analysis were introduced in the regression model. Finally, several hierarchical multiple regression analyses were conducted to test whether depression is associated with multiple lifestyles and personal factors, and more specifically whether personal factors (SES, PAM, SOC-13, HLS-EUQ16, and IPS) moderate the relationship between lifestyles (IPAQ-SF-Walking, IPAQ-SF-Sedentarism, PSQI, and MEDAS) and depression (BDI-II). In the first steps, two variables were included as: one of the lifestyle variables and one of the personal factors. If they accounted for a significant amount of variance in BDI-II, an interaction term between them was created. Next, the interaction term between them was added to the regression model; if it accounted for a significant proportion of BDI-II, we examined the interaction plot in order to establish the direction of the relationship.

Moderation analyses were performed using Hayes’s PROCESS macro (v. 3.2; Hayes, 2018) for IBM SPSS Statistics software (version 25.0; IBM Corp., 2017). Bootstrap resampling (5.000 samples) was used to estimate 95% confidence intervals. Given that heteroscedasticity is common in cross-sectional data and that our sample consisted of less than 250 subjects, all analyses included a correction for heteroscedasticity (HC3; Long and Ervin, 2000). The Johnson-Neyman technique was used to compute the range of significance and simple slopes for the interaction analyses (Hayes, 2018). We reported unstandardized regression coefficients; all analyses were two-tailed and used conventional significance thresholds (*α* = 0.05). The reliability analysis was performed using the R statistical software environment (version 3.6.2; R Core Team, 2019). The descriptive, bivariate, and multivariable analysis were performed using IBM SPSS Statistics software (version 25.0; IBM Corp., 2017).

## Results

### Descriptive and Bivariate Analysis

Firstly, the descriptive analysis is shown in Table 1. Of the 226 participants, 185 were females and 41 were males, and all participants fell between the age range of 20 to 86 years old (mean age = 53.54, SD = 13.41).

**Table 1:** Demographic characteristics, lifestyle variables, and personal factors of the sample.

Variables	Total sample (*n* = 226)	Pearson correlation coefficient with BDI-II	Value of *p*
*Gender*			
Male, *n* (%)	41 (18.1)	0.117	0.080
Female, *n* (%)	185 (81.9)		
*Education*			
None or primary, *n* (%)	87 (38.5)	−0.010	0.879
Secondary or tertiary, *n* (%)	139 (61.5)		
*Occupation*			
Working, *n* (%)	67 (29.6)	0.031	0.644
Not working, *n* (%)	159 (70.4)		
*Marital status*			
With a partner, *n* (%)	126 (55.8)	0.014	0.836
Without a partner, *n* (%)	100 (44.2)		
*Economic level*			
<IMW to 2 IMW, *n* (%)	205 (90.7)	−0.129	0.052
>2 IMW, *n* (*%*)	21 (9.3)		
Age, *years*, *M* (*SD*)	53.54 (13.41)	**−0.147**	**0.027**
IPAQ-SF-Walking (minutes per week), *M* (SD)	212.32 (285.31)	0.053	0.425
IPAQ-SF-Sedentarism (minutes per day), *M* (*SD*)	288.90 (185.25)	**0.136**	**0.042**
PSQI*, M* (*SD*)	11.18 (4.64)	**0.344**	**<0.001**
MEDAS, *M* (*SD*)	6.38 (1.85)	−0.089	0.181
MOS-SS, *M* (*SD*)	12.02 (3.03)	**−0.193**	**0.004**
SES, *M* (*SD*)	169.38 (47.10)	**−0.412**	**<0.001**
PAM*, M (SD)*	40.43 (6.12)	**−0.333**	**<0.001**
SOC-13, *M* (*SD*)	46.31 (12.78)	**−0.546**	**<0.001**
HLS-EUQ16, *M* (*SD*)	31.55 (7.14)	**0.150**	**0.024**
IPS, *M* (*SD*)	28.01 (6.95)	**0.309**	**<0.001**

*IMW, Interprofessional Minimum Wage; BDI-II, Beck II Self-Applied Depression Inventory; IPAQ-SF, Physical Activity Questionnaire-Short Form; PSQI, Pittsburgh Sleep Quality Index; MEDAS, Mediterranean Diet Adherence Screener; MOS-SS, Medical Outcomes Study Social Support Survey; SES, Self-Efficacy Scale; PAM, Patient Activation Questionnaire; SOC-13, Sense of Coherence questionnaire; HLS-EUQ16, Health Literacy Europe Questionnaire; and IPS, Irrational Procrastination Scale*. Significant differences (*p* ≤ 0.05) are highlighted in bold font.

Table 1 shows the results of the bivariate analysis of depressive symptomatology, sociodemographic variables, lifestyle variables, and personal factors. There is a significant relationship between level of depression and age (−0.147, *p* = 0.027), sedentarism (minutes seated per day; 0.136, *p* = 0.042), sleep quality (0.344, *p* < 0.001), social support (−0.193, *p* = 0.004), self-efficacy (−0.412, *p* < 0.001), patient activation in their own health (−0.333, *p* < 0.001), sense of coherence (−0.546, *p* < 0.001), health literacy (0.150, *p* = 0.024), and procrastination (0.309, *p* < 0.001). Adherence to a Mediterranean diet was not significant (−0.089, *p* = 0.181), neither were the minutes spent walking per week (0.053, *p* = 0.425), nor the rest of sociodemographic variables.

### Multivariate Analysis

Regarding the multivariate analysis, once the stepwise regression eliminated the weakest correlated variables, the remaining variables are shown in Table 2. Low sense of coherence (*β* = −0.172; *p* < 0.001), poor sleep quality (*β* = 0.179; *p* = 0.008), low patient activation (*β* = −0.119; *p* = 0.019), and sedentarism (more minutes seated per day; *β* = 0.003; *p* = 0.025) are predictors of having more severe depressive symptoms. This model explains 33% of the overall variance [*R*^2^ adjusted = 0.336, *F*(4,221) = 29.507, *p* < 0.001].

**Table 2:** Regression model of the BDI-II scores with lifestyle variables and personal factors as predictors.

Model	Unstandardized coefficients		Standardized coefficients				Collinearity statistics	
	B	*SE*	Beta	*t*	Value of *p*	95% CI for B	Tolerance	VIF
(Constant)	34.297	2.331		14.711	**0.000**	[29.703, 38.892]		
SOC-13	**−0.172**	0.026	−0.421	−6.603	**0.000**	[−0.223, −0.120]	0.724	1.381
PSQI	**0.179**	0.067	0.159	2.675	**0.008**	[0.047, 0.310]	0.834	1.199
PAM	**−0.119**	0.050	−0.140	−2.366	**0.019**	[−0.218, −0.020]	0.844	1.184
IPAQ-SF-Sedentarism	**0.003**	0.002	0.123	2.258	**0.025**	[0.000, 0.006]	0.992	1.008

Significant differences (*p* ≤ 0.05) are highlighted in bold font. CI, confidence interval. Dependent Variable: Beck II Self-Applied Depression Inventory (BDI-II). IPAQ-SF, Physical Activity Questionnaire-Short Form; PSQI, Pittsburgh Sleep Quality Index; PAM, Patient Activation Questionnaire; and SOC-13, Sense of Coherence questionnaire.

### Moderation Analysis

Regression coefficients were obtained for the variables that showed significant results in the bivariate analysis. In each model, one of the significant lifestyle variables (PSQI or IPAQ-SF-Sedentarism) and one of the significant personal factors (SES, PAM, SOC-13, HLS-EUQ16, or IPS) were included. Next, as all the models were significant, the interaction term between them was created and added to the regression model. At this point, only the interaction between SES and PSQI was significant, so that was the only moderation worth exploring. So, to test whether SES moderates the relationship between PSQI and depression (BDI-II), hierarchical multiple regression analyses were conducted. In the first step, two variables were included as: SES and PSQI. These two variables accounted for a significant amount of depression, *R*^2^ = 0.226, *F*(2, 223) = 32.517, *p* < 0.001. The interaction term between them was added to the regression model which accounted for a significant proportion of the variance in BDI-II, Δ*R*^2^ = 0.014, Δ*F*(1, 222) = 4.07, *p* < 0.001. However, the effect only showed a tendency *b* = 0.0026, *t*(222) = 1.942, *p* = 0.053 (Table 3). As this moderation was not significant, the interaction plot was not examined to establish the direction of the relationship.

**Table 3:** Linear regression analysis of personal factors, lifestyle variables, and the interaction between them on depression.

Variables	*R* ^2^	*F*(2, 223)	Value of *p*	Δ*R*^2^	Δ*F*(1, 222)	Value of *p*	Interaction
SES and PSQI	0.226	32.517	**<0.001**	0.014	4.07	**<0.001**	*b* = 0.0026, *t*(222) = 1.942, *p* = 0.053
PAM and PSQI	0.198	27.464	**<0.001**	0.009	2.47	0.118	–
SOC-13 and PSQI	0.317	51.686	**<0.001**	0.007	2.35	0.127	–
HLS-EUQ16 and PSQI	0.129	16.570	**<0.001**	0.009	2.37	0.125	–
IPS and PSQI	0.180	24.446	**<0.001**	0.003	0.77	0.381	–
MOS-SS and PSQI	0.134	17.231	**<0.001**	0.136	0.523	0.470	-
SES and Sedentarism	0.189	25.916	**<0.001**	0.000	0.009	0.926	–
PAM and Sedentarism	0.128	16.418	**<0.001**	0.004	1.115	0.292	–
SOC-13 and Sedentarism	0.310	50.190	**<0.001**	0.000	0.074	0.789	–
HLS-EUQ16 and Sedentarism	0.042	4.88	**<0.001**	0.006	1.374	0.242	–
IPS and Sedentarism	0.114	14.309	**<0.001**	0.004	1.000	0.318	–
MOS-SS and Sedentarism	0.066	7.833	**<0.001**	0.070	1.033	0.311	–

Dependent Variable: Beck II Self-Applied Depression Inventory (BDI-II). IPAQ-SF, Physical Activity Questionnaire-Short Form; PSQI, Pittsburgh Sleep Quality Index; MEDAS, Mediterranean Diet Adherence Screener; MOS-SS, Medical Outcomes Study Social Support Survey; SES, Self-Efficacy Scale; PAM, Patient Activation Questionnaire; SOC-13, Sense of Coherence questionnaire; HLS-EUQ16, Health Literacy Europe Questionnaire; and IPS, Irrational Procrastination Scale. Significant differences (*p* ≤ 0.05) are highlighted in bold font.

## Discussion

This study aimed to analyze the association between the severity of the depression, lifestyle patterns (physical exercise, sleep, and diet), and personal factors related to health behavior (self-efficacy, activation in their own health, sense of coherence, health literacy, and procrastination).

Regarding the multivariate analysis, data showed that having a low sense of coherence, poor sleep quality, low patient activation, and sedentarism were predictors of having higher depressive symptomatology. Findings as such add to the body of evidence that some lifestyles and personal factors are related to depression. In line with our results, it has been widely shown that sense of coherence is inversely related to depression, presenting a protective capacity against depressive symptoms (Plata-Muñoz et al., 2004; Skärsäter et al., 2009; Anyfantakis et al., 2015; López-Martínez et al., 2019). In line with other studies, patient activation in their own health is associated with better mental health (McCusker et al., 2016) and is negatively correlated with depression (Ngooi et al., 2017). Depressive symptoms are typically accompanied by emotions of helplessness and poor quality of life, which are then related to low patient activation scores (Magnezi et al., 2014). A sedentary lifestyle is recognized as a risk factor for depression (Porras-Segovia et al., 2019) since sedentary people tend to dedicate less time to physical exercise or social activities (Zhai et al., 2015). Sleep quality has also been shown to have significant weight in explaining depression. Sleep has been linked to depression in multiple studies since a lack of sleep is one of the most frequent symptoms that appear in depression (Wang et al., 2019).

The bivariate analysis revealed that individuals with more depressive symptoms had lower age, and a lower score in social support, self-efficacy, patient activation in their own health, sense of coherence, and lower levels of health literacy. In addition, depressive symptoms were related to a more sedentary lifestyle, poor sleep quality, and more procrastination. However, no significant relationship was found with regard to the adherence to a Mediterranean diet, nor with the minutes of walking per week. Regarding adherence to a Mediterranean diet, this non-significant association could be due to the fact that the mean score obtained in our sample was 6.38. In some studies, to consider that there is optimal adherence to a Mediterranean diet pattern, the result needed to be greater than 9 (Salvatore-Benito et al., 2019; Gregório et al., 2020). This low score has been found in several studies that show how Spain and other Mediterranean-based countries are moving away from Mediterranean diet patterns (Godoy-Izquierdo et al., 2021). Regarding minutes spent walking per week, this non-significant association could be due to the fact that the mean of minutes spent walking per week in our sample was 212.32, which is more or less the amount recommended (150 min per week of moderate aerobic physical activity or a minimum of 75 min per week of vigorous aerobic activity; World Health Organization, 2010). Our sample had an adequate amount (in minutes) of physical exercise, but the predominant physical activity was walking, which is the one with the lowest intensity. Therefore, although all physical exercise provides health benefits, the frequency, intensity, and duration of the exercise have a significant influence on its benefits (World Health Organization, 2010). This relationship requires further research as the questionnaire used (IPAQ-SF) has inherent shortcomings (i.e., individuals may have had conceptual difficulty distinguishing between terms such as vigorous or moderate physical activity; people may have had certain limits recalling weekly activities; and there may have been overestimations regarding the amount of physical activity done. In any case, IPAQ-SF reliability and validity have been rigorously verified in many countries, and it is widely used in current international research (Aibar et al., 2016)).

Furthermore, this study also aimed to analyze whether personal factors moderate the relationship between lifestyles and depression. These moderation analyses were not significant. This means that in the present sample, the relationship between the lifestyles tested (sleep quality and sedentarism) and depression does not change according to the value of the different moderators (i.e., personal factors). The interrelationship between lifestyles, personal factors, and depression should be studied further, for example, in larger sample sizes where there may be more power to detect moderation effects, by testing other types of analysis such as mediation, and/or using longitudinal data.

The present SDA study provides data for gaining knowledge and understanding into the relationships between lifestyle, personal factors, and depression. Given the results that some lifestyle and personal factors are related to depression, some programs that modify lifestyle and personal factors might be beneficial in reducing symptoms of depression. This would be in line with recent meta-analyses of RCTs of Lifestyle Modification Programs (LMPs) suggesting that LMPs might be effective in mitigating depressive symptoms (Gómez-Gómez et al., 2020; Wong et al., 2021).

## Strengths

This type of research, in which various topics about healthy lifestyles and personal factors are analyzed together in their association with depression (sleep quality, physical exercise, adherence to a Mediterranean diet, self-efficacy, patient activation in their own health, sense of coherence, health literacy, and procrastination), is scarce. This study adds to the body of evidence that some lifestyles and personal factors are related to depression. In addition, the profile of the participants corresponded with the profile of those who generally attend PHCs consultations, as the majority of patients have depression, and this type of patient is usually treated in PHCs, with only a small percentage of them being referred to a specialist.

## Limitations

Even though SDAs complement primary data collection, could be a suitable starting point for some research, and are a cost-effective way of describing the current situation (McCaston, 2005), they have some limitations. For example, we are not able to make causal inferences (Wickham, 2019), and the associations identified might be difficult to interpret (Wang and Cheng, 2020). Because this is an exploratory SDA of an RCT, there were no sample size estimates or *p*-value adjustments. So, the findings must be interpreted with caution and should only be regarded as preliminary signs that should be studied further.

## Conclusion

Depression is a common pathology worldwide. Lifestyle and personal factors are related to depressive symptomatology. Our findings reveal that sense of coherence, the patient’s activation level, sedentarism, and sleep quality are associated with depression. Further research is needed regarding adherence to Mediterranean diet, minutes walking per week and the interrelationship between lifestyles, personal factors, and depression.

## Data Availability Statement

The raw data supporting the conclusions of this article will be made available by the authors, without undue reservation.

## Ethics Statement

The studies involving human participants were reviewed and approved by Research Ethics Committee of Aragón (CEICA, PI18/286) and the Research Ethics Committee of the Balearic Islands (IB3950/19 PI). The patients/participants provided their written informed consent to participate in this study.

## Author Contributions

BO-B, MS-R, and CN: conceptualization and supervision. AA-L: data curation, software, and visualization. AA-L and EG: formal analysis and methodology. BO-B: funding acquisition, project administration, and validation. AA-L, BO-B, MS-R, and CN: investigation. AA-L and BO-B: writing—original draft. EG, MS-R, and CN: writing—review and editing. All authors contributed to the article and approved the submitted version.

## Funding

This work was supported by Carlos III Health Institute grant number PI18/01336. The funders have no role in study design, data collection and analysis, decision to publish, and manuscript preparation. The funding body will conduct an audit trail once a year.

## Conflict of Interest

The authors declare that the research was conducted in the absence of any commercial or financial relationships that could be construed as a potential conflict of interest.

## Publisher’s Note

All claims expressed in this article are solely those of the authors and do not necessarily represent those of their affiliated organizations, or those of the publisher, the editors and the reviewers. Any product that may be evaluated in this article, or claim that may be made by its manufacturer, is not guaranteed or endorsed by the publisher.
